# An Obscure and Interesting Case of Bright’s Disease

**Published:** 1884-03

**Authors:** 


### Clinical Reports.

### An Obscure and Interesting Case of Bright's Disease Diagnosed by an Ophthalmoscopic Examination—Nephritic Retinitis Present—Death Six Days Afterwards.

Reported by Dr. Grove.

Some of the diseases pertaining to the kidneys are so insidious in their progress that all methods of examination which can discover the origin or direct the physician to the source of mischief should be borne in mind. It is not the purpose of the writer, at this time, to consider the relation of albuminuria in general with nephritic retinitis, but rather to show very briefly the assistance which can be rendered by an ophthalmoscopic examination in masked cases of Bright's disease. When anasarca exists, it is surely a common-sense practice in all such cases to examine the urine. If, then, albuminuria is present, the attention of the physician is directed to the proper channel and some progress, at least, in diagnosis is the result. But in a small per cent. of cases, no œdema even is present and the patient may complain only of cepalalgia, anorexia and a "weak stomach." It is under just such circumstances that the medical attendant is often befogged, and depending on symptoms alone fails to discover, for a time at least, their essential cause.

Schweigger of Berlin, in his lectures on the ophthalmoscope, says, in substance, that in those cases where anasarsa is absent, the patient is often thought to be suffering from biliousness. In eight to ten per cent. of the cases of Bright's disease, there is more or less deterioration of vision, and the amblyopia is often the first cause to alarm the patient. The presence of nephritic retinitis is discovered with the ophthalmoscope and discloses a condition of affairs unknown to the patient and not even suspected by the medical adviser. It is true certain tumors of the brain, more especially of the cerebellum, cause a form of retinitis very much like that depending on Bright's disease. The history of the case allows one, however, readily to differentiate, while the examination of the urine lends an additional corroborating evidence. Why the retina should sympathize with certain affections of the kidneys, and such a characteristic retinitis be produced, is unknown. One theory is that the urea which fails to be excreted interferes with the nutrition of the retina, causing the peculiar degeneration of its tissue. On the other hand, the tension of the aortic current which is so decidedly increased in most cases is given as the explanation. The conditions attending various cases render either idea tenable.

The following case is reported, since it seems to possess some features of unusual interest. On the first day of Jan., 1884, Mr. B—— consulted Dr. Storck, and gave the following history: Aged 37, and unmarried; muscular and always enjoyed good health. During the past six months has been unduly exerting himself in his business as "agent on the road." Has used intoxicating drinks rather freely and smoked excessively. In the early part of last July suffered for the first time from a severe frontal headache. Has had these "aches" occasionally since, but never considered them of any moment.

At present, Jan. 1st, the patient complains of general malaise, some frontal headache and anorexia. Has a great thirst and drinks much water. Can retain but little solid food in stomach, and then with considerable distress. Has no pain bodily but rather restless. Complains of poor vision and occasionally has diplopia. The diagnosis made was nervous prostration complicated with dyspepsia. Tonics, sedatives, and stomachic correctives were prescribed, and the patient advised to consult the writer in regard to his eyes. Jan. 17th, 9 A. M., I saw patient for first time in office. Found lids and all portions of conjunctiva in apparently normal condition. Pupil was rather small, which prevented a satisfactory examination by ophthalmoscope. Wished to dilate pupil with atropine solution, but patient preferred to wait until evening. The field of vision was not restricted for either eye. Tension of eye-balls normal.

V. O. D. $\frac{20}{100}$, but after decided effort $\frac{20}{80}$.

V. O. S. $\frac{20}{200}$, but after decided effort $\frac{20}{100}$.

7 P. M., one drop of an atropia sulph. solution (gr. i to aquæ ℥i) instilled and patient advised to call to-morrow morning.

Jan. 18th, 10 A. M. Patient called for examination, expecting afterwards to visit place of business. The pupil was about twice as large as normal, and the examination with ophthalmoscope presented an exact picture of advanced retinitis albuminuria. On close inspection of orbital conjunctiva a slight effusion was noticed, and the lids, perhaps, were somewhat puffy. There was no œdema in any part of the body and no pain or soreness over the region of kidneys on pressure. The V. O. U. had now decreased to that extent so that fingers were only visible ten feet away. The patient complains of becoming easily exhausted after slight exertion and inability to retain anything on stomach. Bowels move once a day, but only about one-half the normal amount of urine passed daily. The patient was advised to return home immediately and "take to his bed." A sample of his urine was obtained and the examination showed the following:

Acid in reaction, light straw color—sp. gr. 1.010, albumen present, which, after settling, showed a bulk of about one-third of urine in test-tube. No sugar or hyaline casts found.

The result of the examination was reported to Dr. Storck, the family physician, and during the afternoon we visited the patient together. We found him restless but complaining of no pain—temp. 101½°, pulse 120 and strong. On pressure over right kidney some soreness present.

Perfect rest in bed was enjoined and hot poultices around the body over the region of the kidneys ordered. ℞. A pill of resina podophyll., calomel and digitalis. Also, bromidum potassium as sedative.

Jan. 19th. Rested better last night and can retain some nourishment. Pulse 100, temp. 100°. Bowels opened four to five times in past twenty-four hours and about eight ounces of urine passed in same time; skin dry; ordered hot bath.

The urine acid—straw color—sp. grav. 1.010; albumen present.

Jan. 20th, 10 A. M. Pulse 80, temp. 99°. Some pain for the first time on pressure over region of kidneys. No œdema of extremities or elsewhere. ℞. Potassæ acetatis gr. ten every three hours.

5 P. M. Pulse 80, temp. 98½°. Patient very restless and will not retain external applications in place. Stomach will not tolerate medicine or nourishment. The urine acid and sp. gr. 1.010. The same amount of urine voided as on previous days and the same amount of albumen present.

Jan. 21st, A. M. Dr. Wyckoff was called in consultation. Emesis of all nourishment and medicine continues. V. O. U.=fingers at seven feet. ℞. Bismuth gr. v, *calomel* gr. 1-10 dry on tongue every two hours Also, potassæ acetatis gr. v with infus, Digitalis ʒi every two hours.

P. M. Stomach in better condition, for some nourishment is retained. Temp. normal, pulse 90. The urine acid sp. gr. 1.010 and albumen to the same amount as yesterday present.

Jan. 22d, A. M. Rested fairly during night. The vision remains about the same.

P. M. Complains of feeling dizzy for the first time, and a numb sensation in extremities of fingers. The amount of urine voided is not increased. It is of a very light color, acid reaction and sp. gr. 1.005. Pulse 88 and temp. 98½°.

Jan. 23d, A. M. Dr. Wyckoff again present; patient restless, and stomach very irritable. The urine very scanty and the last sample voided at 2 A. M. It is acid in reaction; sp. grav. 1.010. There is much pain on pressure over kidneys, light straw color. Not so much albumen present as in yesterday's specimen.

At 11 A. M., sedatives and nourishment were given per rectum.

4 P. M., patient has had two hours of sleep, but on awakening complains of dizziness. Skin is moist, pulse 120, temp. 98½°. Bladder contains no urine.

11 P. M., Dr. Storck passed catheter and found no urine.

Jan. 24th, 5 A. M. Death occurred without convulsions. No post mortem, unfortunately, was obtained. The urine voided for the past four days, and especially that passed on the 22d, did not have the odor of normal urine, and after standing for a few days even the smell was not unpleasant. This was owing, of course, to the retention of the greater portion of the urine in the body, which did not allow by decomposition the ammonia compounds to be found in the urine voided.

